# Assessment of Expression of *SOCS* Genes in Acquired Immune-Mediated Polyneuropathies

**DOI:** 10.3389/fimmu.2021.712859

**Published:** 2021-07-19

**Authors:** Mohammad Taheri, Somayeh Sangseifid, Pariya Shahani, Mohammad Mahdi Eftekharian, Shahram Arsang-Jang, Soudeh Ghafouri-Fard

**Affiliations:** ^1^ Skull Base Research Center, Loghman Hakim Hospital, Shahid Beheshti University of Medical Sciences, Tehran, Iran; ^2^ Department of Immunology, School of Medicine, Hamadan University of Medical Sciences, Hamadan, Iran; ^3^ Department of Cellular Molecular Biology, Faculty of New Sciences, Medical Tehran Branch, Islamic Azad University, Tehran, Iran; ^4^ Neurophysiology Research Center, Hamadan University of Medical Sciences, Hamadan, Iran; ^5^ Cancer Gene Therapy Research Center, Zanjan University of Medical Science, Zanjan, Iran; ^6^ Department of Medical Genetics, School of Medicine, Shahid Beheshti University of Medical Sciences, Tehran, Iran

**Keywords:** acquired immune-mediated polyneuropathies, Guillain-Barré syndrome, CIDP, AIDP, suppressor of cytokine signaling

## Abstract

Acquired immune-mediated polyneuropathies are classified to some subtypes among them are acute and chronic inflammatory demyelinating polyradiculoneuropathies (AIDP and CIDP). These two conditions share some common signs and underlying mechanisms. Based on the roles of *Suppressor of cytokine signaling* (*SOCS*) genes in the modulation of immune system reactions, these genes might be involved in the pathogenesis of these conditions. We evaluated expression of *SOCS1-3* and *SOCS5* genes in the leukocytes of 32 cases of CIDP, 19 cases of AIDP and 40 age- and sex-matched controls using real time PCR method. The Bayesian regression model was used to estimate differences in mean values of genes expressions between cases and control group. Expression levels of *SOCS1* and *SOCS2* were significantly lower in male patients compared with controls. This sex-specific pattern was also observed for *SOCS3* down-regulation. Based on the area under curve values in Receiver Operating Characteristics (ROC) curve, diagnostic powers of *SOCS1*, *SOCS2*, *SOCS3* and *SOCS5* genes in the mentioned disorder were 0.61, 0.73, 0.68 and 0.58, respectively. Expression of none of genes was correlated with age of enrolled cases. The current study shows evidences for participation of *SOCS* genes in the pathophysiology of acquired immune-mediated polyneuropathies.

## Introduction

Immune-mediated neuropathies embrace a variety of peripheral nerve disorders which can be classified according to the course of signs evolution, principal engagement of motor/sensory fibers, dispersal of signs and paraclinical factors (1, 2). Two types of these neuropathies are Guillain-Barré syndrome (GBS) and chronic inflammatory demyelinating polyradiculoneuropathy (CIDP) (2). GBS can be classified to acute IDP (AIDP) and axonal forms with variable incidences in different parts of the world (2). AIDP accounts for more than 90% of GBS patients in Western world (3). The pathologic events during evolution of GBS are commonly triggered by environmental factors such as infections or vaccination that stimulate abnormal immune responses, interruption in the blood–nerve barrier and demolition of myelin sheaths and/or axons (4–6). Aberrant immune responses in GBS are mostly mediated by T helper cell-dependent induction of macrophages (7). While several studies have highlighted the role of Th1 cells producing proinflammatory cytokines in this condition, some pathogenic events in the GBS cannot be explained by Th1/Th2 imbalance, so other types of T cells such as Th17 and regulatory T cells might also been involved in this process (8).

CIDP is another type of immune mediated neuropathies which is described by the involvement of proximal and distal motor/sensory fibers. This disorder can have relapsing or progressive courses (9). Several parts of immune regulatory mechanisms including antibodies against autoantigens, complement, T lymphocytes and macrophage cells are involved in triggering abnormal immune response in CIDP (9).

Suppressors of cytokine signaling (SOCS) proteins represent a family of proteins which are located inside the cell and partake in the regulation of immune cell responses to cytokines (10). As negative regulators of the cytokine–JAK–STAT pathway, these proteins participate in diverse immune-related and pathological processes (10). Aberrant expressions of SOCS proteins have been linked with some immunological diseases such as rheumatoid arthritis (11), inflammatory bowel disease (12), allergic responses (13) and multiple sclerosis (14).

Since SOCS proteins partake in the control of immune cascades, we evaluated expression of *SOCS1-3* and *SOCS5* genes in the peripheral blood cells of individual affected by immune-mediated neuropathies and healthy controls to find their possible role in the pathophysiology of this immune-related condition. The reason for selection of *SOCS1-3* and *SOCS5* genes was their aberrant expression in bipolar disorder, a neurological disorder in which aberrant immune responses are involved in the pathogenesis (15). We also aimed at assessment of their possible application as biomarkers for this disorder. This field has been explored by a number of recent studies that focused on identification of differentially expressed molecules between GBS patients and healthy subjects. These novel biomarkers can be applied for identification of the pathophysiology of this disorder, primary diagnosis, intervention, and judgment about the prognosis (16).

## Materials and Methods

### Patients and Normal Subjects

The current investigation was conducted on blood samples obtained from 32 cases of typical CIDP (10 females and 22 males), 19 cases of AIDP (6 females and 13 males) and 40 healthy subjects (11 females and 29 males) (**Table 1**). Patients were recruited during April 2019-April 2020 from Imam Hossein hospital, Tehran, Iran. AIDP and CIDP patients were diagnosed based on guidelines described by European Federation of Neurological Societies (17) and National Institute of Neurological Disorders and Stroke (18). Besides, electrophysiological criteria were used for diagnosis of AIDP cases (19). Patients had no obvious sign or symptom of disorder at the time of blood sampling and did not take any immune modulatory drug in at least 2 weeks prior to sampling (20). All of AIDP and CIDP cases were in remission. None of study participants had recent or chronic infection, neoplasm or any systemic disorder which alters immune responses. The study protocol was approved by ethical committee of Shahid Beheshti University of Medical Sciences (IR.SBMU.MSP.REC.1398.855). All study participants signed the informed consent forms.

**Table 1:** Demographic data of enrolled people in the study.

Variables	Patients	Healthy subjects
Female/Male [no. (%)]	16 (32%)/35 (68%)	11 (27.5%)/29 (72.5%)
Age (mean ± standard deviation, Y)	36.2 ± 2.7	35.3 ± 2.4
Age range (Y)	18-85	19-81
Age of onset (mean ± standard deviation, Y)	31.42 ± 2.79	–
Disease duration (mean *±* standard deviation, Y)	4.57 ± 3.19	–

### Expression Assays

Peripheral blood samples were gathered from patients and controls in EDTA-containing tubes. Total RNA was extracted from blood samples using the commercial kit provided by GeneAll Company after red blood cells lysis (Seoul, South Korea). Next, cDNA was produced from 50-100 ng of RNA (BioFact™, Seoul, South Korea). The RealQ Plus 2x Master Mix for Probe (Ampliqon) and StepOnePlus™ RealTime PCR System (Applied Biosystems, Foster city, CA, USA) were used for real time PCR. **Table 2** shows the features of primers and probes. PCR program comprised an initial activation phase for 5 minutes at 94° C, and 40 cycles at 94 °C for 10 seconds and 60 °C for 40 seconds. Total reaction volumes were 20 µL containing 4 μl of cDNA, 3.5 μl double distilled water, 10 μl of Master Mix 2×, 250 nM and 900 nM concentrations of probe and each primer, respectively. Cycle threshold (Ct) values of genes were corrected for efficiency of amplification. Relative expression of genes in each sample was estimated based on calculation of Ln [Efficiency^ΔCT] values. Efficiency values for *SOCS* genes and *HPRT1* were between 1.7 and 1.9.

**Table 2:** The detailed features of primers.

Gene name	Primer and probe sequence	Product size
*HPRT1*	F: AGCCTAAGATGAGAGTTC	88
	R: CACAGAACTAGAACATTGATA	
	FAM -CATCTGGAGTCCTATTGACATCGC- TAMRA	
*SOCS1*	F: TGGCCCCTTCTGTAGGATGG	109
	R: GGAGGAGGAAGAGGAGGAAGG	
	FAM- TGGCCCCTTCTGTAGGATGG- TAMRA	
*SOCS2*	F: ACGCGAACCCTTCTCTGACC	99
	R: CATTCCCGGAGGGCTCAAGG	
	FAM -CTCGGGCGGCCACCTGTCTTTGC-TAMRA	
*SOCS3*	F: GTGGAGAGGCTGAGGGACTC	111
	R: GGCTGACATTCCCAGTGCTC	
	FAM- CACCAAGCCAGCCCACAGCCAGG- TAMRA	
*SOCS5*	F: GTGACTCGGAAGAGGATACAACC	91
	R: CTAACATGGGTATGGCTGTCTCC	
	FAM- CGCTGCTTCTGCCTCCGTGACTGC- TAMRA	

### Statistical Methods

The Bayesian regression model using Laplace prior family was used to estimate differences in genes expressions between cases and control group. This method permitted control of the effects of gender, age, and assessment of group*gender interaction effects. Models compared using Watanabe-Akaike information criterion and leave-one-out cross-validation. The Receiver Operating Characteristic (ROC) regression model was used to estimate best cut-off values of expression levels of genes for distinguishing cases from controls. The figures of the Bayesian model were appraised using Hamiltonian Monte Carlo chains with 6000 iterations and 1000 warm-up in RStan C++ library. The optimal cut-off values in ROC curve analysis were determined using the Youden index (J) method. The pROC, Stan, loo, and shynistan packages were used in R 3.6.1 software. The Hybrid Monte Carlo algorithm was used to yield more efficient estimates through the posterior distribution. The model convergence was checked by R-hat and Gelman-Rubin diagnostics available in Shynistan. P values and 95% credible interval (95% CrI) were calculated. P values were computed from the quantile regression model using the bootstrap method.

## Results

### Information About Study Participants

The study included 32 cases of CIDP and 19 cases of AIDP. Demographic data of these individuals are summarized in **Table 2**.

### Expression Assays

**Figure 1:**
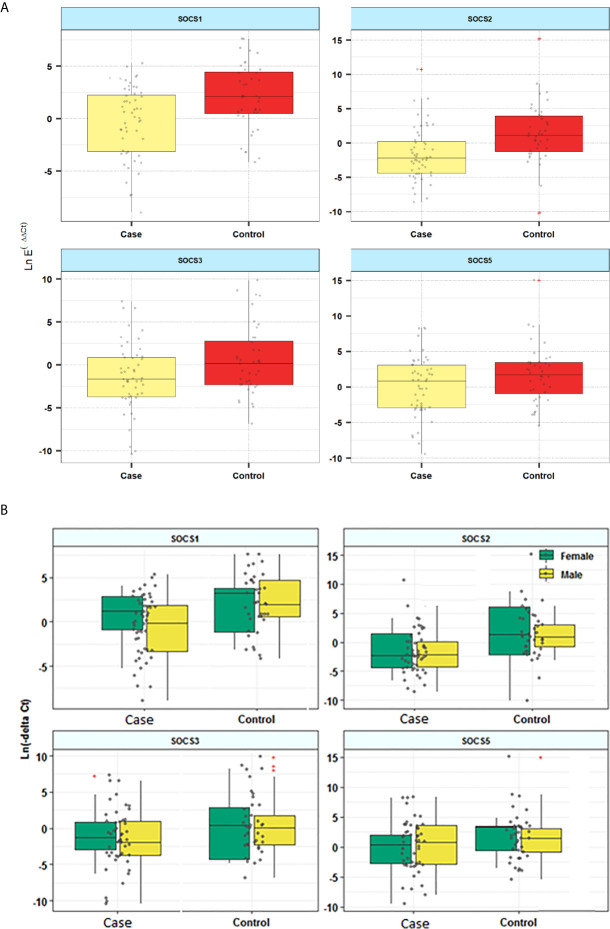
Relative expression of SOCS genes in patients and controls **(A)** and based on the gender of study participants **(B)**.

Expression levels of *SOCS1* and *SOCS2* were significantly lower in patients compared with controls (Posterior Beta=-2.18, P=0.029; Posterior Beta=-3.19, P<0.0001, respectively). When assessing expression levels of these genes in distinct sex-based subgroups, differences in their expressions were significant in male subgroups but not female subgroups. Expression of *SOCS3* was significantly lower in male patients compared with male controls (Posterior Beta=-3.44, P=0.037). However, expression of this gene was not different between female subclasses (Posterior Beta=-0.87, P=0.528). Expression of *SOCS5* was not different between cases and controls or between sex-based subgroups of them. **Figure 1** and **Table 3** show the details of assessment of expression of *SOCS* genes between cases and control subjects. There was no remarkable difference in expression of any *SOCS* gene between AIDP and CIDP patients.

**Table 3:** Relative expression of *SOCS* genes in patients and controls.

		*SOCS1*				*SOCS2*				*SOCS3*				*SOCS5*			
	Variable	Posterior Beta	SE	P-value	95% CrI	Posterior Beta	SE	P-value	95% CrI	Posterior Beta	SE	P-value	95% CrI	Posterior Beta	SE	P-value	95% CrI
Total	Group (Case/Control)	-2.18	0.78	0.029	[-3.71, -0.7]	-3.19	0.79	<0.0001	[-4.76, -1.65]	-1.35	0.76	0.061	[-2.85, 0.16]	-1.33	0.79	0.692	[-2.85, 0.25]
	Gender	-0.92	0.74	0.18	[-2.34, 0.57]	0.16	0.9	0.934	[-1.69, 1.94]	0.05	0.95	0.711	[-1.85, 1.96]	-0.04	0.9	0.454	[-1.8, 1.81]
	Age	-0.01	0.02	0.855	[-0.06, 0.04]	0.01	0.02	0.409	[-0.03, 0.04]	0.02	0.02	0.335	[-0.02, 0.07]	0.04	0.03	0.015	[-0.01, 0.09]
	Group*Gender	-0.98	1.49	0.826	[-3.74, 2.18]	-1	2.06	0.986	[-5.15, 2.89]	-1.14	2.24	0.504	[-5.28, 3.24]	2.17	1.67	0.443	[-1.01, 5.59]
Male	Group (Case/Control)	-2.46	0.96	0.047	[-4.27, -0.51]	-3.44	0.84	<0.0001	[-5.19, -1.82]	-3.44	0.84	0.037	[-5.19, -1.82]	-0.75	1.07	0.684	[-2.8, 1.36]
	Age	-0.01	0.03	0.872	[-0.06, 0.04]	0.01	0.02	0.449	[-0.03, 0.05]	0.01	0.02	0.723	[-0.03, 0.05]	0.05	0.04	0.233	[-0.03, 0.11]
Female	Group (Case/Control)	-1.69	1.34	0.229	[-4.37, 0.79]	-1.69	1.34	0.389	[-4.37, 0.79]	-0.87	1.99	0.528	[-4.98, 2.77]	-2.26	1.31	0.062	[-5.23, 0.01]
	Age	-0.02	0.04	0.583	[-0.1, 0.06]	-0.02	0.04	0.783	[-0.1, 0.06]	0.04	0.05	0.432	[-0.06, 0.14]	0.02	0.04	0.681	[-0.06, 0.1]

Subsequently, we assessed differences in the expression of *SOCS* genes in AIPD cases versus CIDP cases (**Table 4**). Expression of *SOCS1* was significantly higher in AIDP cases compared with CIDP cases. When assessing gene expression in sex-based subgroups, differences were significant only in male subgroups. Expressions of other *SOCS* were not different between AIPD and CIDP cases.

**Table 4:** Relative expression of *SOCS* genes in AIPD cases versus CIDP cases.

	*SOCS1*				*SOCS2*				*SOCS3*				*SOCS5*			
Group	Posterior Beta	SE	P-value	95% CrI	Posterior Beta	SE	P-value	95% CrI	Posterior Beta	SE	P-value	95% CrI	Posterior Beta	SE	P-value	95% CrI
Total	3.816	0.88	0.001	[1.98, 5.45]	0.809	1.06	0.419	[-1.21, 2.97]	2.009	1.23	0.442	[-0.23, 4.76]	0.013	1.16	0.684	[-2.25, 2.34]
Male	4.266	0.92	<0.0001	[2.48, 6.05]	0.458	1.31	0.413	[-2, 3.13]	5.023	1.76	0.046	[1.17, 7.83]	-0.089	1.58	0.721	[-3.05, 3.15]
Female	2.51	2.03	0.13	[-1.34, 6.65]	4.461	3.13	0.63	[-2.15, 10.29]	1.464	2.82	0.4	[-3.89, 6.77]	2.279	1.59	0.655	[-1.07, 5.54]

Additionally, expression of *SOCS* genes was compared between AIDP cases and controls (**Table 5**). Expression of *SOCS1* was higher in AIDP cases versus controls (Posterior beta=4.779, P value<0.0001). Similarly, expressions of SOCS2 and SOCS3 were higher in AIDP cases compared with controls (Posterior beta=3.765, P value=0.005 and Posterior beta=2.757, P value=0.036, respectively). However, expression of *SOCS5* was not different between AIDP cases and controls.

**Table 5:** Relative expression of *SOCS* genes in AIDP cases versus controls.

	*SOCS1*				*SOCS2*				*SOCS3*				*SOCS5*			
Group	Posterior Beta	SE	P-value	95% CrI	Posterior Beta	SE	P-value	95% CrI	Posterior Beta	SE	P-value	95% CrI	Posterior Beta	SE	P-value	95% CrI
Total	4.779	0.87	<0.0001	[3.05, 6.45]	3.765	1.1	0.005	[1.62, 6.04]	2.757	1.2	0.036	[0.58, 5.43]	1.359	1.03	0.655	[-0.62, 3.45]
Male	5.178	0.98	<0.0001	[3.33, 7.25]	3.855	1.24	<0.0001	[1.39, 6.31]	5.917	1.73	0.032	[2.05, 8.51]	0.723	1.48	0.866	[-2.03, 3.84]
Female	-0.007	0.05	0.168	[-0.1, 0.09]	0.002	0.07	0.332	[-0.14, 0.14]	0.054	0.06	0.451	[-0.06, 0.18]	0.022	0.04	0.295	[-0.07, 0.11]

### ROC Curves

**Figure 2:**
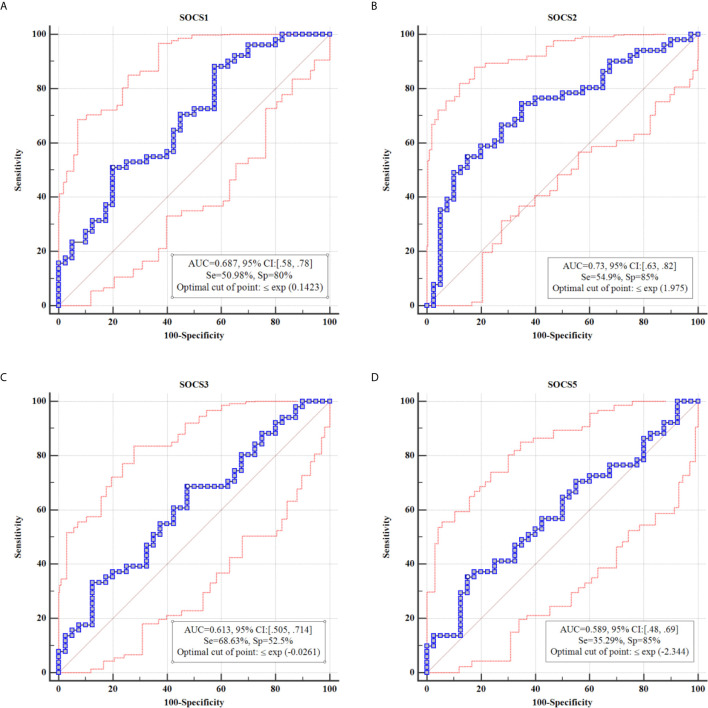
Results of ROC curve analysis for *SOCS1* **(A)**, *SOCS2* **(B)**, *SOCS3* **(C)** and *SOCS5* **(D)**, respectively.

Based on the AUC values, diagnostic powers of *SOCS* genes in the mentioned disorder were 0.61, 0.73, 0.68 and 0.58, respectively. **Figure 2** shows the results of ROC curves assessments.

**Figure 3:**
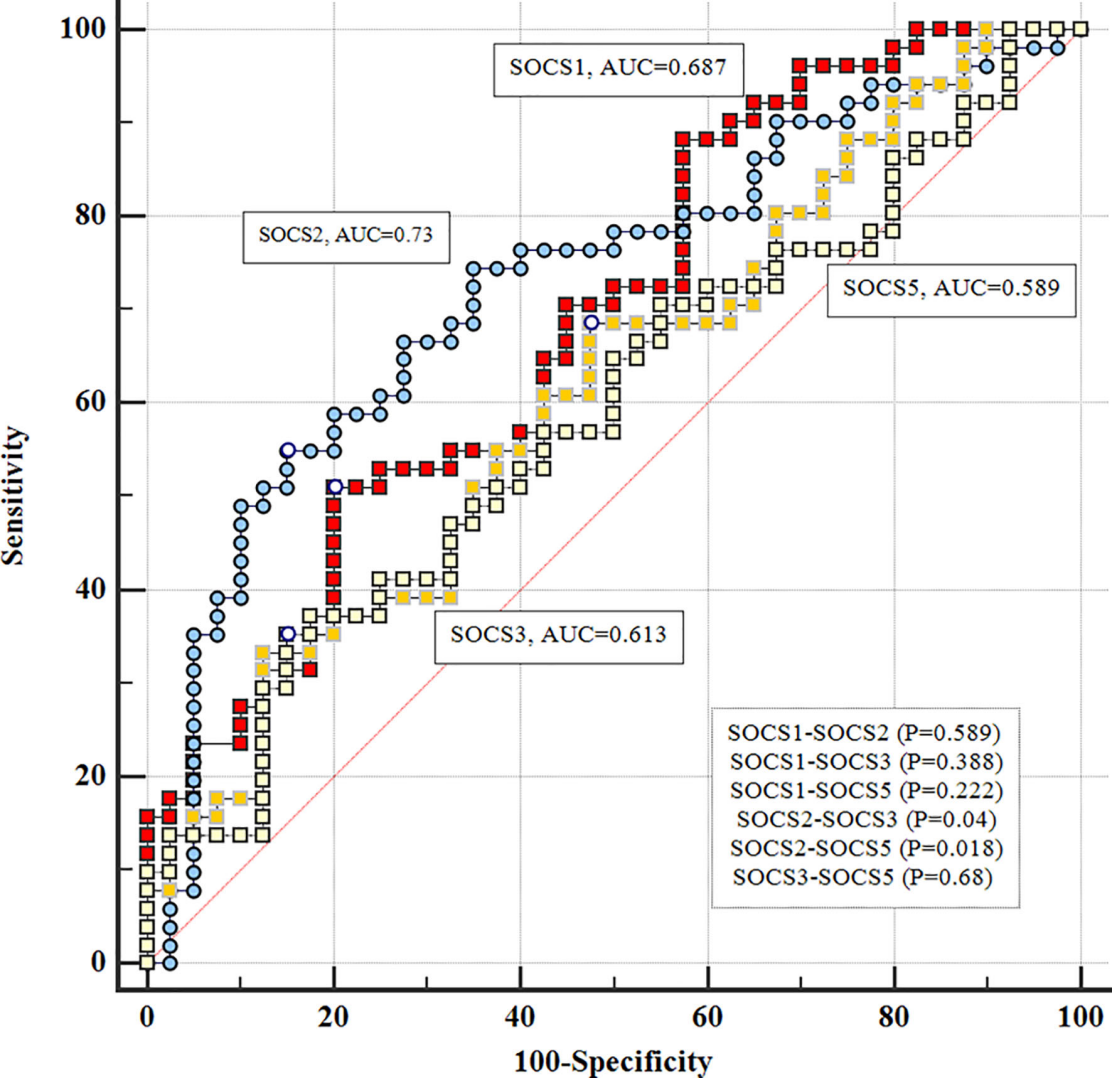
Comparison of diagnostic power of *SOCS* genes in patients.

There were significant differences in diagnostic power of *SOCS2* and *SOCS3* genes (P=0.04) and *SOCS2* and *SOCS5* (P=0.018) (**Figure 3**).

**Figure 4:**
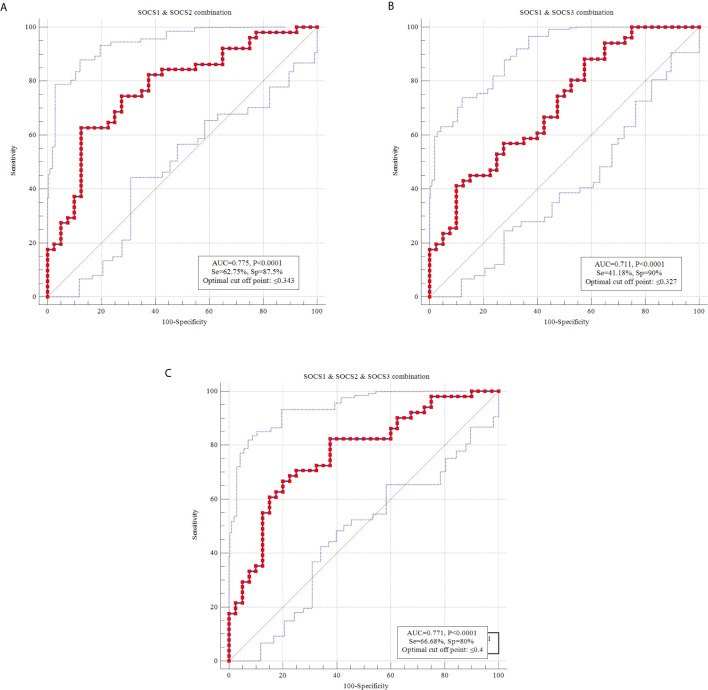
ROC curves using combination of transcript amounts of *SOCS1* and *SOCS2* **(A)**, *SOCS1* and *SOCS3* **(B)** and *SOCS1-3* **(C)**.

Then, we combined ROC curves of pairs of *SOCS* genes and *SOCS1-3* genes together (**Figure 4**). Combination of *SOCS1* and *SOCS2* genes provided the best AUC value (AUC=0.775, sensitivity=62.75%, Specificity=87.5%).

### Correlation Analysis

Remarkable correlations were demonstrated between expressions of *SOCS2* and *SOCS3* (r=0.55, P<0.0001), *SOCS2* and *SOCS5* (r=0.48, P<0.0001), and *SOCS5* and *SOCS3* (r=0.52, P<0.0001). Expression levels of *SOCS* genes were not correlated with age of either patients or healthy subjects.

## Discussion

We examined expression of *SOCS* genes in the samples of peripheral blood cells obtained from AIDP and CIDP patients versus healthy individuals. These two autoimmune conditions have common clinical symptoms, histologic signs and similar therapeutic options (immunosuppressive therapy) (21). The role of aberrant immune responses in these types of neuropathies has been vastly investigated. Among the well-appreciated underlying mechanisms are activation of cellular adhesion molecules that permit transmission of autoreactive T cells and B cells across the blood–nerve barrier, activation of macrophages and enhancement of their phagocytic activity by T lymphocytes, secretion of inflammatory cytokines, nitric oxide, reactive oxygen species and proteases (5, 22). SOCS proteins partake in the regulation of many aspects of immune responses through inhibiting the Jak/STAT signaling pathway (23). Jak-STAT pathway is active in some immune cells including macrophages at a basal level and is robustly activated during innate responses (24). Based on the prominent pathogenic role of macrophages in the immune-related neuropathies, SOCS proteins are possible molecules that could modulate aberrant immune responses in the immune-related neuropathies. However, it is worth mentioning that SOCS proteins are not specifically expressed by macrophages. Therefore, their expression would not necessarily reflect macrophage activation. Moreover, SOCS proteins might have specific functions. For instance, SOCS1 has a role in the development of acute/chronic myeloid leukemia, glioblastoma and Barrett’s adenocarcinoma. SOCS2 partake in the pathogenesis of ovarian cancer, acromegaly associated colonic polyps, osteoarthritis and type 2 diabetes. SOCS3 is involved in the pathobiology of prostate cancer, ulcerative colitis, breast cancer and atopic asthma/dermatitis. Finally, SOCS5 contribute in the pathogenesis of uveitis and thyroid cancer (25). We detected lower expressions of *SOCS1* and *SOCS2* in patients compared with healthy subjects. SOCS1 has been shown to be implicated in the pathogenesis of some immune-related disorders. Lack of Socs1 in a murine model of autoimmune arthritis has led to considerable increase in joint inflammation and destruction (26). Moreover, down-regulation of SOCS1 expression has a role in guiding the pro-inflammatory M1 role of macrophages by activating the JAK/STAT pathway (27). Sosc2 has been shown to improve recovery process of traumatic brain injury in mice through modulation of neuroinflammatory response and stimulation of a more anti-inflammatory setting through alerting M1/M2 macrophages ratio (28). Thus, the observed down-regulation of *SOCS2* in patients with immune-related neuropathies is in line with the role of M1 proinflammatory macrophages in GBS pathogenesis.

When assessing expression levels of these genes in distinct sex-based subgroups, differences in their expressions were significant in male subgroups but not female subgroups. Although studies regarding the role of sex hormones of expression of SOCS genes are few, a previous study has shown activation of SOCS2 expression by estradiol (29). Thus, lack of difference in expression of *SOCS2* between female patients and female controls might be at least partially explained by the regulatory effects of estradiol on its expression. Alternatively, as the sample size of females is considerably smaller than males, the significant finding in males (but not females) might be explained by the differences in the sample sizes. In other words, there might be not really enough age-matched cases/controls to segregate subjects by their sex, i.e. there were not enough female cases to provide meaningful comparison.

In addition, we compared expression of *SOCS* genes between AIDP cases and CIDP cases and detected higher expression of *SOCS1* in male AIDP cases compared with male CIDP cases. Yet, based on the small sample size in this subgroup analysis, the power of this analysis is limited. Thus, we propose conduction of further similar studies in larger sample sizes to find whether the functional role of SOCS genes/proteins is different in CIDP and AIDP.

Expression of *SOCS3* was significantly lower in male patients compared with male controls. However, expression of this gene was not different between female subgroups. SOCS3 has a selective role in inhibition of IL-6 signaling, restricting its ability to inhibit LPS signaling (10). Lack of Socs3 gene in macrophages and neutrophils has increased Th1 activity and secretion of inflammatory cytokines including TNF-α, IL-1, IFN-γ and IL-6 (30). Consequently, our observation regarding lower levels of *SOCS3* in patients is in harmony with the detected phenotype in Socs3-devoid mice. Lack of difference in expression of this gene between female subgroups is best explained by the small sample size of this subgroup.

We also assessed the diagnostic power of these genes. Based on the area under curve values in ROC curve, diagnostic powers of *SOCS1*, *SOCS2*, *SOCS3* and *SOCS5* genes in immune-related neuropathies were 0.61, 0.73, 0.68 and 0.58, respectively. Although none of them is regarded as sensitive or specific biomarker of this disorder, they might be incorporated in putative diagnostic panels for this immune-related condition to predict immune status of patients. However, this speculation should be appraised in future investigations. Combination of *SOCS1* and *SOCS2* genes has enhanced the diagnostic power and yielded the AUC value of 0.775. Previous studies have reported diagnostic value of a number of molecules in GBS. For instance, Li et al. have demonstrated up-regulation of haptoglobin and heat shock protein 70, while down-regulation of cystatin C in cerebrospinal fluid (CSF) of GBS patients. Their results indicated the biomarker roles for these proteins for early GBS diagnosis, though these proteins could not differentiate AIDP and acute motor axonal neuropathy (16). Other studies have suggested a number of infection-/immune-/blood-nerve barrier and the blood-CSF barrier, and peripheral nervous system damage-related biomarkers. However, the clinical applications of several of these suggested biomarkers have been limited by the expense of the discovery method, invasiveness of the needed procedure and low sensitivity/specificity (31). Based on the results of ROC curves, individual *SOCS* genes do not differentiate well between patients and controls. However, combination of these genes might result in better values. Thus, further studies are needed to suggest more sensitive biomarkers for this disorder.

Significant correlations were detected between expressions of *SOCS2* and *SOCS3*, *SOCS2* and *SOCS5*, and *SOCS5* and *SOCS3*. Previous studies have shown the role of SOCS2 in regulation of the protein levels of SOCS1 and SOCS3 (32, 33). SOCS2 has specifically increased SOCS3 degradation (33). However, the observed direction of correlation between these two SOCS members in the current study is not in accordance with this regulatory effect. Consequently, our observation indicates a more complicated interactive network between *SOCS* genes which should be identified in future studies.

Expression of none of genes was correlated with age of enrolled persons. This finding is in agreement with the results of our recent study of *SOCS* genes expressions in breast cancer patients which revealed independence of expression of these genes from age (34).

Taken together, the current study provides evidences for participation of *SOCS* genes in the pathophysiology of CIDP/GBS and necessitates conduction of future functional studies to clarify the underlying mechanism. However, as RNA abundance does not necessarily correlate with protein abundance, we state lack of assessment of SOCS proteins as a limitation of our study. Thus, for translation of the results of study in clinical application, it is necessary to address this point. Besides, we state lack of assessment of genes expression at the active phase of disorder as another limitation of our study. Further studies are needed to test whether these conclusions are valid.

## Data Availability Statement

The original contributions presented in the study are included in the article/supplementary material. Further inquiries can be directed to the corresponding author.

## Ethics Statement

The study protocol was approved by ethical committee of Shahid Beheshti University of Medical Sciences (IR.SBMU.MSP.REC.1398.855). The patients/participants provided their written informed consent to participate in this study.

## Author Contributions

MT and SG-F wrote the draft and revised it. SA-J analyzed the data. SS, ME, and PS performed the experiment. All authors contributed to the article and approved the submitted version.

## Funding

The current study was supported by a grant from Shahid Beheshti University of Medical Sciences.

## Conflict of Interest

The authors declare that the research was conducted in the absence of any commercial or financial relationships that could be construed as a potential conflict of interest.
